# APOBEC3A suppresses cervical cancer via apoptosis

**DOI:** 10.7150/jca.89044

**Published:** 2023-10-16

**Authors:** Zishuai Li, Haiwei He, Xiangyu Ren, Yifan Chen, Wenbin Liu, Rui Pu, Letian Fang, Yiwei Shi, Donghong Liu, Jiayi Zhao, Zheyun Niu, Mingjuan Xu, Guangwen Cao

**Affiliations:** 1 Department of Epidemiology, Second Military Medical University, Shanghai, 200433, China.; 2 Department of Obstetrics and Gynecology, the 1st Affiliated Hospital, Second Military Medical University, Shanghai 200433, China.; 3 Department of Hepatic Surgery, the 3rd Affiliated Hospital, Second Military Medical University, Shanghai, 200438, China.; 4 Shanghai East Hospital, Key Laboratory of Arrhythmias, Ministry of Education, Tongji University School of Medicine Tongji University, Shanghai 200120, China.; 5 Shanghai Key Laboratory of Medical Bioprotection, Shanghai, 200433, China.; 6 Key Laboratory of Biological Defense, Ministry of Education, Shanghai, 200433, China.

**Keywords:** Cervical cancer, apolipoprotein B mRNA-editing enzyme catalytic 3 A (APOBEC3A), proliferation, apoptosis, outcomes

## Abstract

**Background:** Family members of Apolipoprotein B mRNA-editing enzyme catalytic 3 (APOBEC3) play critical roles in cancer evolution and development. However, the role of APOBEC3A in cervical cancer remains to be clarified.

**Methods:** We used bioinformatics to investigate APOBEC3A expression and outcomes using The Cancer Genome Atlas (TCGA)-cervical squamous cell carcinoma and endocervical adenocarcinoma (CESC) dataset, GTEx, and GSE7803. Immunohistochemistry was then used to identify APOBEC3A's expression pattern. We performed Cell Counting Kit-8, wound-healing, Transwell, and flow cytometry assays to measure proliferation, migration, invasion, and apoptosis, respectively, using the SiHa and HeLa cell lines transfected with APOBEC3A. BALB/c nude mice were used to investigate the effects of APOBEC3A* in vivo*. The phosphorylated gamma-H2AX staining assay was applied to measure DNA damage. RNA sequencing (RNA-Seq) was applied to explore APOBEC3A-related signaling pathways.

**Results:** APOBEC3A was more significantly expressed in cancer tissues than in adjacent normal tissues. Higher expression of APOBEC3A was associated with better outcomes in TCGA-CESC and GTEx. Immunohistochemistry showed that the expression of APOBEC3A was significantly higher in cancer tissues than in normal tissues. Transfection experiments showed that APOBEC3A inhibited proliferation, upregulated S-phase cells, inhibited migration and invasion, induced DNA damage, and promoted apoptosis. Overexpression of APOBEC3A inhibited tumor formation in the mouse model. RNA-seq analysis showed that ectopic expression of APOBEC3A inhibited several cancer-associated signaling pathways.

**Conclusions:** APOBEC3A is significantly upregulated in cervical cancer, and higher expression of APOBEC3A is associated with better outcomes. APOBEC3A is a tumor suppressor whose overexpression induces apoptosis in cervical cancer.

## Introduction

Cervical cancer is a common malignant tumor in women worldwide 1. According to the American Cancer Society, cervical cancer is the second most common cancer-related death among females aged 20 to 39 2. In China, age-standardized mortality has increased in the past decade 3. Chronic infection with high-risk human papillomavirus (HPV) promotes the progression from pre-cancerous lesions to benign cervical dysplasia and cervical intraepithelial neoplasia (CIN) and finally to cervical cancer 4.

Apolipoprotein B mRNA-editing enzyme catalytic (APOBEC) is a family of cytidine deaminases that converts cytosines in DNA and RNA to uracil, resulting in DNA/RNA mutations or damage 5. Deep sequencing demonstrated that APOBEC mutations are critical in HPV-induced carcinogenesis and are prevalent in cervical cancer tissues and cancer cell lines 6-9. APOBEC3B expression in cervical cancer is associated with HPV-18 infection 10 and is often associated with cell proliferation, migration, and cell cycle progression in cervical cancers, except for gastric-type cervical adenocarcinoma 11-13. APOBEC3A overexpression leads to deamination and degradation of transfected exogenous plasmid DNA, suggesting that APOBEC3A may mediate the clearance of HPV DNA in persistently infected cells 14.

APOBEC3A is the enzyme with the highest catalytic activity among APOBEC3 proteins and is a significant source of APOBEC-signature mutations 15. APOBEC3A-induced cytidine deamination-associated somatic mutations are enriched in cervical cancer genomes, where the C-to-T mutational signature predominates 16, 17. C-to-T or G-to-A mutations also exist in the mitochondrial DNA of cervical cancer cells 18. Uracil-DNA glycosylase (UNG) is a critical molecule in the nucleic acid base excision repair pathways, which can recognize and excise the C-to-U mutation induced by APOBECs, triggering the hydrolysis of the nucleic acid chain for mismatch repair 19. We suggested that the imbalance between APOBEC3B and UNG caused by chronic non-resolving inflammation may promote hepatocellular carcinoma 20. APOBEC3A has potent deamination activity in breast cancer cell lines and is strongly associated with mutational burden in breast tumors, suggesting that APOBEC3A is a significant driver of genomic mutations in cancer of distinct histotypes 21.

In contrast, APOBEC3A is a protective marker and a prognostic biomarker for predicting survival and immunotherapy response in ovarian cancer 22. However, the roles of APOBEC3A in cervical cancer are unclear. Therefore, we explored the APOBEC3A expression pattern and its effects on biological behaviors such as proliferation, apoptosis, invasion, and migration in cervical cancer.

## Materials and Methods

### Bioinformatics analysis

The RNA-Seq data of cervical squamous cell carcinoma and endocervical adenocarcinoma (CESC), adjacent normal samples (TCGA-CESC), and normal cervical samples (GTEx database) were downloaded from UCSC Xena (https://xenabrowser.net/). The TCGA-CESC and GTEx cohorts were combined to form a larger cohort (TCGA and GTEx) consisting of 13 normal cervical tissues and 294 CESC samples. The GSE7803 dataset was obtained from the GEO database (https://www.ncbi.nlm.nih.gov/geo/). All downloaded patients had no other malignant tumors or diseases. No patient had undergone adjuvant treatment before surgery. The clinical phenotypes were grouped and analyzed. Gene set enrichment analysis (GSEA) plots were generated using the R packages *GSEABase*, *DESeq2*, *clusterProfiler*, and *enrichplot*. Survival plots were drawn using the R packages *survivor* and *survminer*, and box plots were drawn using the R package *ggplot2*. All analyses used the R Foundation for Statistical Computing (2021) version 4.1.1.

### Participants

We used 77 cases from the First Affiliated Hospital of Second Military Medical University between January 2014 and January 2017. These included 38 patients with cervical cancer, 16 with CIN who underwent total hysterectomy or cervical conization, and 23 histologically confirmed normal patients who underwent total hysterectomy for symptomatic uterine fibroids or adenomyosis. All patients were hospitalized for surgery; no other malignant tumors were combined. No patient had undergone adjuvant treatment before surgery. Cervical formalin-fixed paraffin-embedded tissues were collected from each participant. The Second Military Medical University Ethics Committee approved the study, and all patients provided written informed consent.

### Immunohistochemistry (IHC), quantification, and histoscore

Cervical cancer, CIN, and normal cervical tissues were processed using standard techniques. Anti-APOBEC3A (1:50 dilution; abcam, ab262853, San Diego, CA, USA) was used for IHC staining. Three investigators (Tan XJ, Yu YW, and Lu SL) evaluated the IHC staining independently. APOBEC3A-stained slides were scanned using Aperio ScanScope XT (Leica Biosystems) software, and the images were magnified 40 times for histological scoring, as reported previously 23. In selected areas of the specimen, the Aperio Nuclear Algorithm ranked the immunostaining as negative (-), slightly positive (+), moderately positive (++), or strongly positive (+++). The APOBEC3A H-score was calculated using the linear formula: H-score = 1 × (% slightly positive cells) + 2 × (% moderately positive cells) + 3 × (% strongly positive cells). Two of the three investigators usually reached a close agreement (90%) on H-score. Otherwise, a consensus was reached by discussion.

### Cell culture and plasmids

HeLa, SiHa, and HEK293T cells were purchased from the Cell Bank of the Chinese Academy of Sciences (Shanghai, China). HeLa and HEK293T cells were cultured in Dulbecco's modified Eagle's medium (DMEM) (BasalMedia, Shanghai, China), and SiHa cells were cultured in Minimum Essential Medium (GIBCO, Grand Island, NY, USA). The media were supplemented with 10% fetal bovine serum (Gibco, New York, NY, USA) and 1% penicillin/streptomycin (Bio-light, Shanghai, China). Cell cultures were performed in a humidified 5% CO_2_ environment at 37 °C. Mycoplasma contamination test of cell cultures was performed every 3 months. All experiments were performed with mycoplasma-free cells. The APOBEC3A-expressing vector containing human APOBEC3A coding sequence in the pLenti-CMV-mCherry-PGK-blasticidin expression vector was purchased from Obio Technology (Shanghai, China). The structure diagrams of empty control and APOBEC3A expressing plasmids are shown in Figure S1. The APOBEC3A expressing and empty control plasmids were transfected using a Lipofectamine 3000 kit (Invitrogen, Carlsbad, CA, USA).

### Western blot

Western blotting was conducted as described 24. The primary antibodies were anti-APOBEC3A (dilution 1:500, PA5-78800, Invitrogen) and anti-GAPDH (dilution 1:1000, sc-25778, Santa Cruz, Dallas, TX, USA) rabbit polyclonal antibodies. GAPDH was used as the loading and normalization control. ImageJ software (version 1.51, https://imagej.nih.gov/ij/) was used to quantify the signal strength of the bands. We performed at least three independent experiments.

### Cell Counting Kit-8 (CCK8), cell cycle, and apoptosis assays

A CCK8 kit (Dojindo, Osaka, Japan) was used to assess cell proliferation. Cells were seeded into 96-well plates (Corning Incorporated Coster, Kennebunk, ME) at 2000 cells per well. Every 24 h, 10 µL of CCK8 was added to wells and incubated for 2 h before measuring optical density at 450 nm. For cell cycle assay, cells were digested and fixed at -20 °C in 75% ethanol overnight. Propidium iodide/RNase staining buffer (BD, San Diego, CA, USA) measured the cell cycle distribution using flow cytometry (Merck Millipore, Rockville, MD, USA). ModFit LT software (Verity Software House, Topsham, ME) analyzed the raw data. For apoptosis assay, cells were harvested in 0.25% trypsin without EDTA (Gibco, Grand Island, NY, USA) and resuspended in binding buffer (BD Pharmingen, San Diego, CA, USA), stained with Annexin V-FITC and 7-AAD (BD Pharmingen, San Diego, CA, USA) according to the manufacturer's instructions. A Beckman Coulter CytoFlEX S flow cytometer (Beckman Coulter, Miami, FL) was used to measure apoptosis.

### Wound-healing and Transwell migration/invasion assays

The *in vitro* wound-healing assay was performed to study directional cell migration. Briefly, HeLa and SiHa cells were seeded in six-well plates at 5 × 10^5^ cells/well. After incubation for 24 h, APOBEC3A overexpression and empty control plasmids were transfected using Lipofectamine 3000 kit (Invitrogen). We created a scrape with a 100-µL pipette tip when the monolayers reached confluence. Cells were washed with phosphate-buffered saline, and images of migrated cells were captured at zero and 48 h using a fluorescence microscope (Leica, Germany). The wound-healing rate was analyzed using Image J software. The formula was % Cure Rate = (New Surface - Previous Surface) / New Surface × 100.

Cell invasion and migration were measured using Transwell inserts (Corning, NY, USA). We added 100 µL of Matrigel (Corning) vertically to the bottom center of the upper chamber, incubated at 37 °C for 5 h, and discarded the residual liquid for the invasion assay. The migration assay did not require Matrigel (Corning).

We seeded 4 × 10^5^ cells into the upper chamber with 100 µL serum-free DMEM containing 0.1% bovine serum albumin (Bio-light, Shanghai, China). The lower chamber was filled with 500 µL DMEM containing 10% fetal bovine serum. After incubation for 24 h, the upper chamber was transferred to 500 μL of 4% paraformaldehyde (Bio-light) and fixed at room temperature for 15 min. Then, the upper chamber was transferred to crystalline violet staining solution (Bio-light, Shanghai, China), stained for 20 min, dried with a cotton swab, and photographed under the microscope. Five fields were randomly selected and photographed with a microscope at 10 × magnification. ImageJ software (https://imagej.net/software/imagej/) was used to calculate the cell numbers. Each assay was performed in triplicate.

### Construction of lentivirus and animal experiments

The APOBEC3A-expressing and empty control plasmids were transfected into HEK293T cells with the MD2G packaging plasmid and PAX2 envelope plasmid using a Lipofectamine 3000 kit (Invitrogen). After incubation for 48 h, lentivirus-containing supernatants were harvested, and lentiviral particles were concentrated using Lenti-X (Clontech, CA, USA). SiHa cells were infected with lentivirus for 24 h, and two stable cell strains (SiHa-APOBEC3A and SiHa-Vector) were screened using 10 μg/mL Blasticidin S (Yeasen, Shanghai, China).

We purchased 20 female mice (BALB/c-nude) from Cavens Laboratory Animal (Changzhou, China) to examine the tumorigenicity. Twenty mice were randomly assigned to two groups. SiHa-APOBEC3A cells (5 × 10^6^) or SiHa-Vector cells (5 × 10^6^) were mixed with Matrigel and injected into mice. The tumor volumes were measured twice weekly and calculated as volume = 0.5 × length × width^2^. After 38 days, mice were sacrificed, and xenograft tumors and mice were weighed. Tumors were harvested for hematoxylin-eosin staining. The Ethics Committee of the Second Military Medical University approved the protocol.

### The phosphorylated gamma-H2AX staining assay

HeLa and SiHa cells were seeded into 96-well plates (Corning) at 2000 cells per well. The APOBEC3A-expressing and empty control plasmids were transfected using a Lipofectamine 3000 kit (Invitrogen). After incubated for 48 h, the phosphorylated gamma-H2AX staining were performed using the DNA Damage Assay Kit by γ-H2AX Immunofluorescence (Beyotime Biotechnology, China) following the manufacturer's instructions. The images were taken on a fluorescence microscope (Leica, Germany) with an exposure time of 2s, and analyzed with ImageJ software (https://imagej.net/software/imagej/).

### RNA sequencing (RNA-Seq)

Total RNA was isolated from two cell lines (SiHa-APOBEC3A and SiHa-Vector) using TRIzol (Invitrogen) and preserved at -80 °C. A NanoDrop spectrophotometer (Thermo Fisher Scientific, Waltham, MA, USA) determined the concentration, quality, and integrity. Three micrograms of RNA were used as input material for the RNA sample preparations. Sequencing libraries were generated using a TruSeq RNA Sample Preparation Kit (Illumina, San Diego, CA, USA). RNA-Seq and analysis were performed at Shanghai Personal Biotechnology Co., Ltd. (Shanghai, China).

### Statistical analysis

Analyses were performed using GraphPad Prism version 8.3.0 (GraphPad Software, San Diego, CA, USA). The Student's* t*-test was used to determine differences in continuous variables, while the chi-square test was used to evaluate differences in categorical variables. Transcriptome data and survival analysis were performed using R platform version 4.1.1 (Core Development Team, http://www.r-project.org/). Survival plots were generated using the R packages *survivor* and *survminer*. The box plots were created using the R package *ggplot2*. A two-sided *P* < 0.05 was considered statistically significant for all analyses.

## Results

### APOBEC3A is significantly upregulated in cervical cancer, and higher expression of APOBEC3A is associated with better outcomes

**Figure 1:**
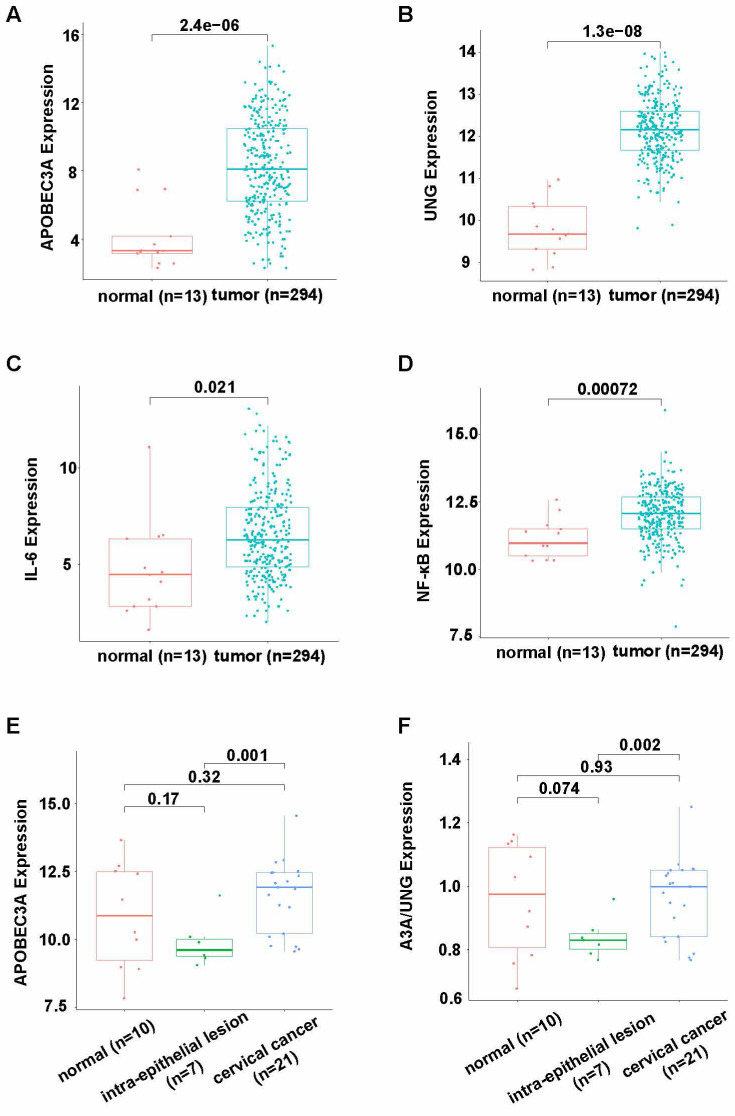
** Differential expression of APOBEC3A and UNG in cervical cancer according to bioinformatics analysis.** (A) APOBEC3A expression level in cervical cancer using data from TCGA and GTEx. (B) UNG expression level in cervical cancer using data from TCGA and GTEx. (C) IL-6 expression level in cervical cancer using data from TCGA and GTEx. (D) NF-κB expression level in cervical cancer using data from TCGA and GTEx. (E) APOBEC3A expression level in cervical cancer using data from GSE7803 dataset. (F) The ratio of APOBEC3A expression level to UNG expression level in GSE7803. UNG: uracil-DNA glycosylase, IL-6: interleukin-6, NF-κB: nuclear factor kappa-B

APOBEC3A and UNG expression levels were significantly higher in cervical cancer than in adjacent normal tissues, according to TCGA and GTEx (*P* < 0.001, Figures 1A and B). The expression of interleukin-6 (IL-6) and nuclear factor kappa-B (NF-κB) was significantly higher in tumor tissues than normal tissues (*P* = 0.021 and *P* = 0.00072, respectively), suggesting more inflammation in tumor tissues than in normal tissues. (Figures 1C and D). According to GSE7803, APOBEC3A expression was significantly higher in cervical cancer than in intraepithelial lesion tissues (*P* = 0.001, Figure 1E). The APOBEC3A to UNG expression level ratio was significantly higher in cervical cancer than in intraepithelial lesion tissues (*P* = 0.002, Figure 1F).

**Figure 2:**
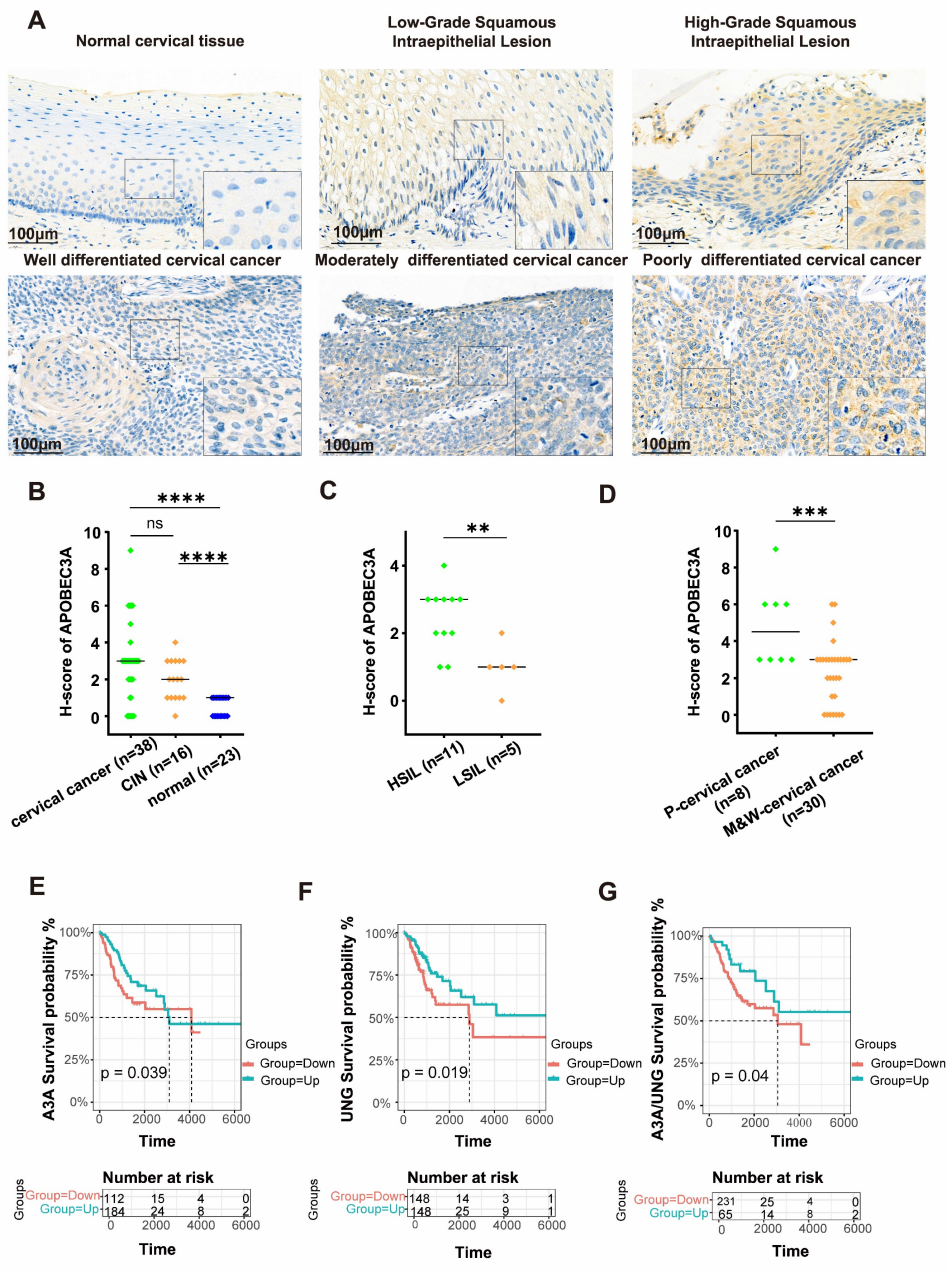
** APOBEC3A expression in cervical cancer by immunohistochemistry and outcomes by bioinformatics analysis.** (A) APOBEC3A protein expression level in normal cervical tissue, CIN, and cervical cancer. (B) APOBEC3A level was higher in cervical cancer than in normal cervical tissues, and APOBEC3A level in CIN was higher than in normal cervical tissues. (C) The level of APOBEC3A is higher in HSIL than in LSIL tissues. (D) The level of APOBEC3A is higher in poorly differentiated cervical cancer than in moderately and well-differentiated cervical cancer. (E) Overall survival analysis for APOBEC3A. (F) Overall survival analysis for UNG. (G) Overall survival analysis for the ratio of APOBEC3A expression level to UNG expression level. *P < 0.05, **P < 0.01, ***P < 0.001, ****p < 0.0001, ns, no significance. HSIL, High-Grade Squamous Intraepithelial Lesion; LSIL, Low-Grade Squamous Intraepithelial Lesion; P-cervical cancer, poorly differentiated cervical cancer; M&W-cervical cancer, moderately and well-differentiated cervical cancer.

Representative IHC images were shown in Figure 2A. IHC revealed that APOBEC3A expression was significantly higher in cervical cancer than in normal cervical tissues (*P* < 0.001), and APOBEC3A expression level in CIN tissues was higher than in normal cervical tissues (*P* < 0.001) (Figure 2B). High-grade squamous intraepithelial lesions (HSILs) had higher APOBEC3A expression than low-grade squamous intraepithelial lesions (LSILs) (*P* < 0.01) (Figure 2C). Poorly differentiated cervical cancer tissues had higher APOBEC3A expression than moderately well-differentiated cervical cancer (*P* < 0.001) (Figure 2D).

We classified lesions above or equal to the median H-score as having high APOBEC3A expression (H-APOBEC3A) and those below the median as having low expression (L-APOBEC3A). High APOBEC3A expression was associated with advanced International Federation of Gynecology and Obstetrics (FIGO) staging (χ² = 12.931, *P* = 0.002), lymph node metastasis (χ² = 4.156, *P* = 0.041), and pathological histological hypofractionation (χ² = 5.911, *P* = 0.015) in cervical cancer patients (Supplementary Table S1). After removing patients containing missing values, we also categorized the TCGA downloaded patients into high APOBEC3A expression (H-APOBEC3A) and low expression (L-APOBEC3A) groups (Supplementary Table S2). The downloaded data did not contain the information of histological grade and tumor size. The results showed that APOBEC3A expression was not associated with FIGO stage (χ² =6.5951, P =0.08599) and lymph node metastasis (χ² = 3.6467, P =0.05618), which were inconsistent with the results of our own cohort. A total of 183 patients containing missing values in the downloaded data were excluded, which creates a serious bias. By comparing the common clinical parameters between our own cohort and the TCGA cohort, we found no significant statistical difference between the two cohorts (Supplementary Table S3).

Higher expression of APOBEC3A and UNG in cervical cancer was associated with better outcomes (*P* = 0.039 and *P* = 0.019, respectively) (Figures 2E and F), according to TCGA. A higher ratio of APOBEC3A to UNG was associated with better outcomes (*P* = 0.04) (Figure 2G).

### The effects of APOBEC3A on cervical cancer progression in *vitro* and in *vivo*

**Figure 3:**
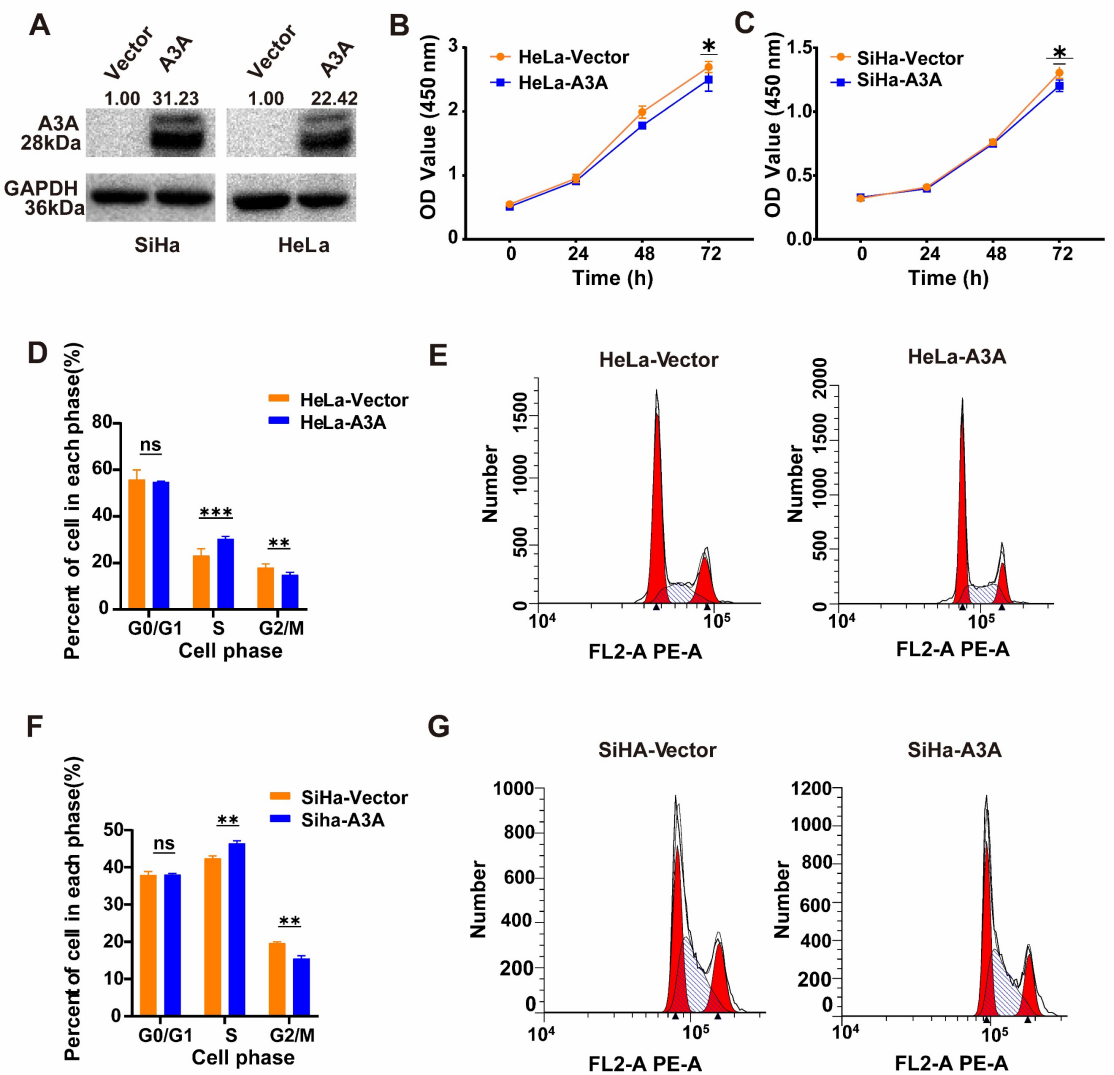
** The effects of ectopic overexpression of APOBEC3A on cervical cancer cell proliferation, cell cycle, and wound healing.** (A) Western blot assay results of APOBEC3A protein. (B) CCK8 assay showed APOBEC3A overexpression inhibits cellular proliferation in HeLa cells. (C) CCK8 assay showed APOBEC3A overexpression inhibits cellular proliferation in SiHa cells. (D) Typical flow cytometric analysis of the cell cycle in HeLa cells. (E) Statistical analysis of cell phase in HeLa cells. (F) Typical flow cytometric analysis of the cell cycle in SiHa cells. (G) Statistical analysis of cell phase in SiHa cells. *P < 0.05, **P < 0.01, ***P < 0.001, ****P < 0.0001, ns, no significance.

Ectopic expression of APOBEC3A in SiHa and HeLa cells was demonstrated using western blots (Figure 3A). The CCK8 assay indicated that the ectopic expression of APOBEC3A significantly inhibited proliferation in HeLa and SiHa cells (Figure 3B and Figure 3C). Flow cytometry results demonstrated that upregulating APOBEC3A led to a significant upregulation of cells at the S-phase and significant downregulation at the G2/M-phase (Figure 3D-3G).

**Figure 4:**
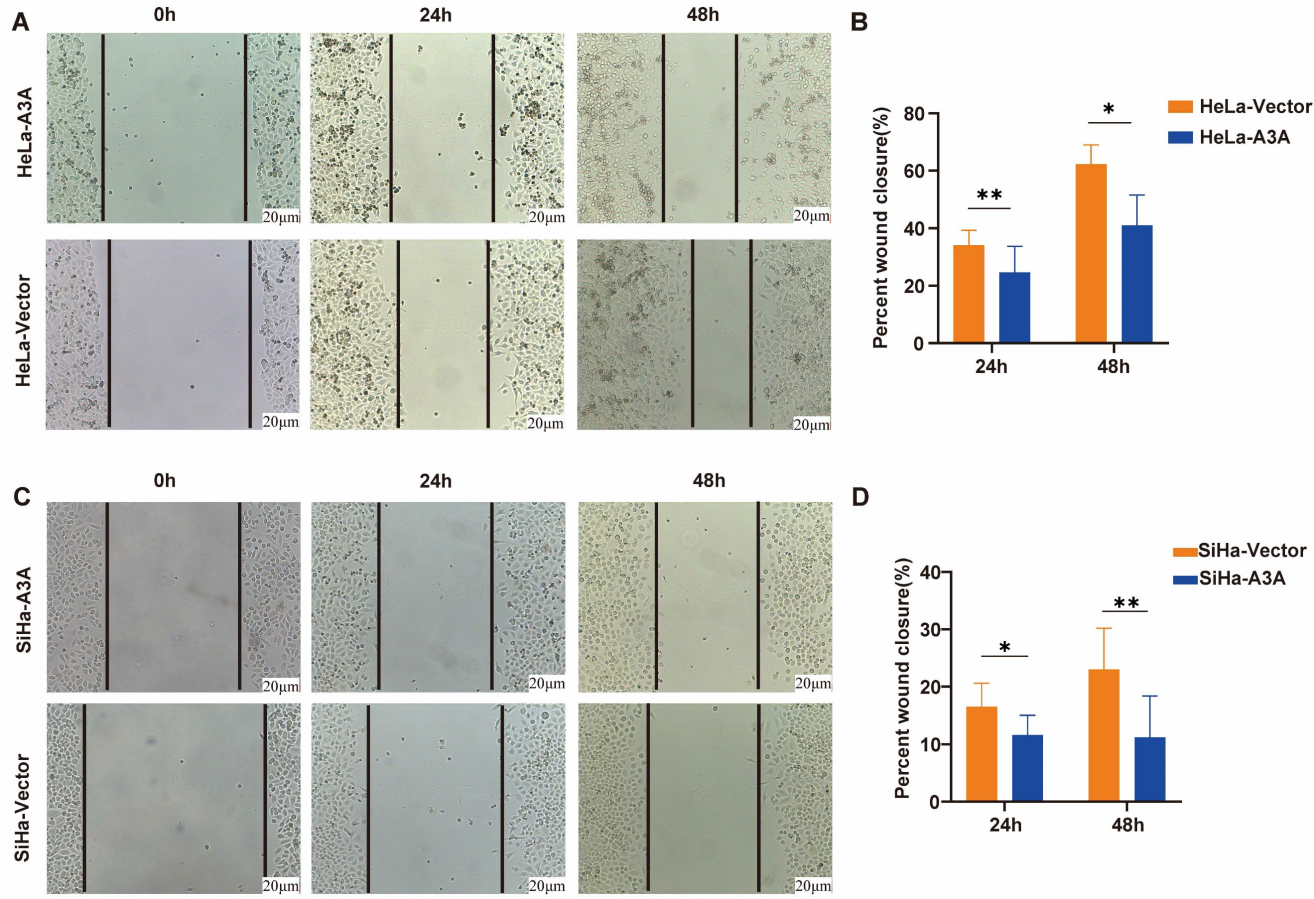
** The effects of ectopic overexpression of APOBEC3A on cervical cancer cell wound healing.** (A) Representative images of the wound-healing assay in HeLa cells. (B) Statistical analysis of wound-healing assay in HeLa cells. (C) Representative images of the wound-healing assay in SiHa cells. (D) Statistical analysis of wound-healing assay in SiHa cells. Vector, transfected with control plasmid; A3A, transfected with APOBEC3A overexpression plasmid; *P < 0.05, **P < 0.01.

**Figure 5:**
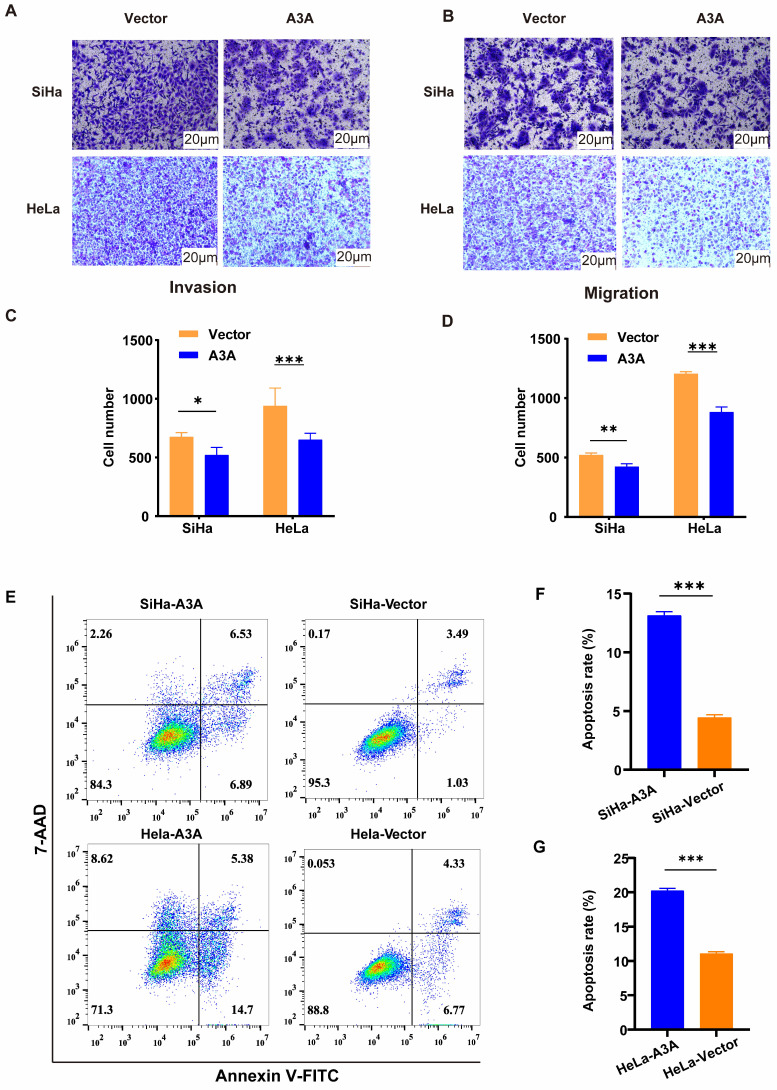
**Ectopic expression of APOBEC3A inhibits migration and invasion and promotes apoptosis of cervical cancer cells.** (A) Representative images of the Transwell invasion assay. (B) Representative images of the Transwell migration assay. (C) Statistical analysis of the Transwell invasion assay. (D) Statistical analysis of the Transwell migration assay. (E) Representative images of flow cytometry. (F) Statistical analysis of apoptosis in SiHa cells. (G) Statistical analysis of apoptosis in HeLa cells. Vector, transfected with control plasmid; A3A, transfected with APOBEC3A overexpression plasmid; *P < 0.05, **P < 0.01, ***P < 0.001.

**Figure 6:**
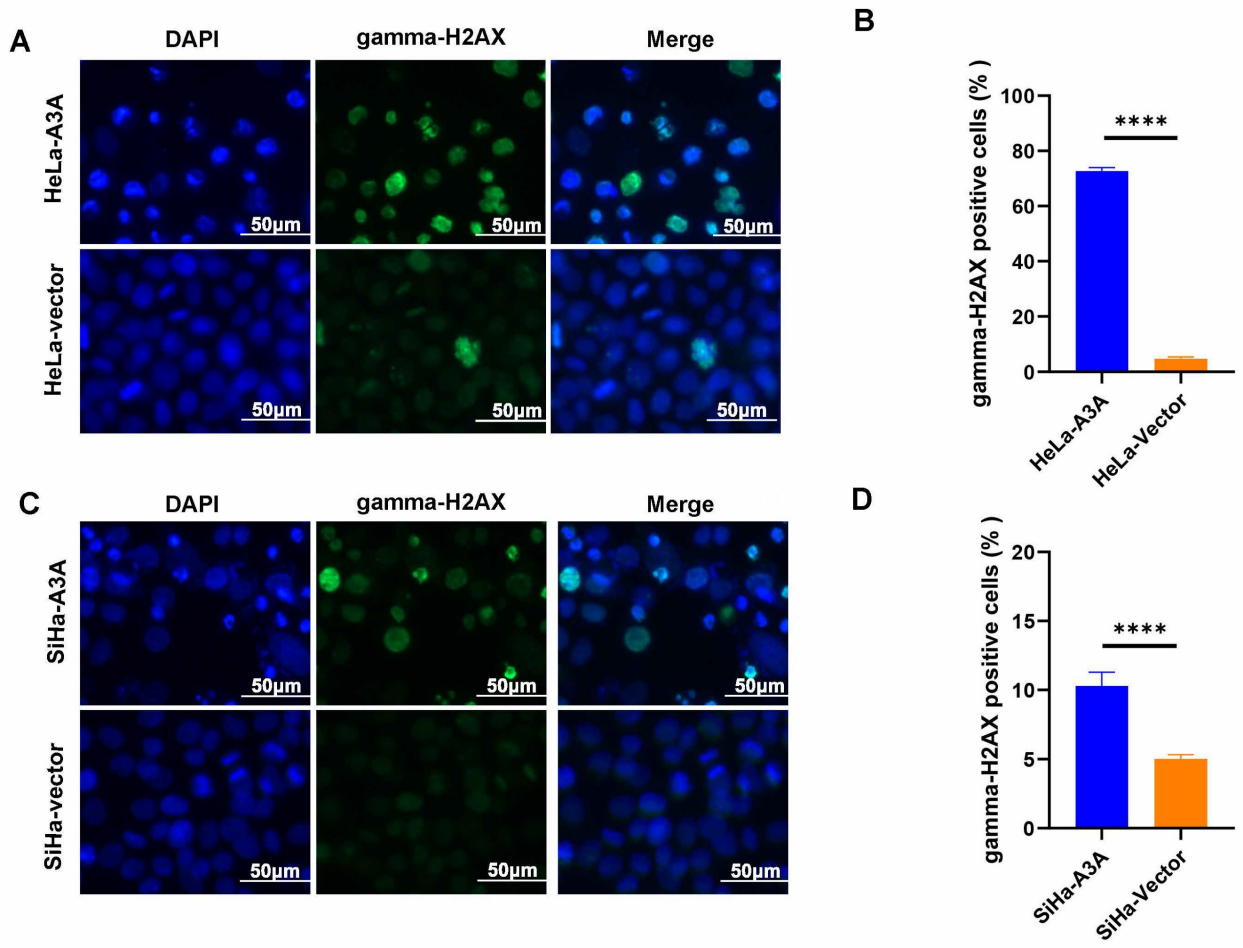
**Ectopic expression of APOBEC3A promotes DNA double strand break of cervical cancer cells.** (A) Representative images of the phosphorylated gamma-H2AX staining assay in HeLa cells. (B) Statistical analysis of phosphorylated gamma-H2AX staining assay in HeLa cells. (C) Representative images of the phosphorylated gamma-H2AX staining assay in SiHa cells. (D) Statistical analysis of phosphorylated gamma-H2AX staining assay in SiHa cells. Vector, transfected with control plasmid; A3A, transfected with APOBEC3A overexpression plasmid; ****P < 0.0001.

Ectopic expression of APOBEC3A inhibited the wound-healing rate at 24 and 48 h in HeLa and SiHa cells (Figure 4). The Transwell assay demonstrated that compared with HeLa-Vector and SiHa-Vector cells, HeLa-APOBEC3A and SiHa-APOBEC3A cells exhibited weaker invasion and migration ability, respectively (Figure 5A-5D). Ectopic expression of APOBEC3A significantly increased cell apoptosis in SiHa and HeLa cells (*P* < 0.01) (Figure 5E-5G). Staining for phosphorylated gamma histone 2AX (γ-H2AX) revealed that the ectopic expression of APOBEC3A significantly increased numbers of γ-H2AX-positive cells in in HeLa and SiHa cells (*P* < 0.0001) (Figure 6), suggesting that overexpression of APOBEC3A is cytotoxic.

**Figure 7:**
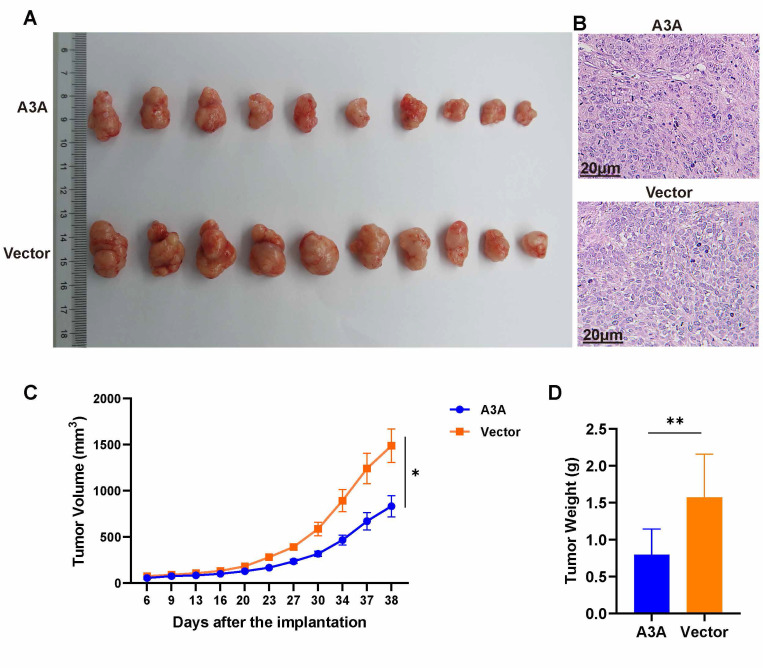
**APOBEC3A inhibits tumor formation in* vivo*.** (A) Tumor size. (B) Hematoxylin-eosin staining of tumor tissues. (C) Tumor volume growth curve. (D) Tumor weights. Vector, infected with control lentivirus; A3A, infected with APOBEC3A overexpression lentivirus; *P < 0.05, **P < 0.01.

The effects of APOBEC3A overexpression were also investigated *in vivo*. Tumor volumes and weights were smaller in the APOBEC3A overexpression group than in the control group (Figure 7A). At day 38, the average tumor size in the SiHa-APOBEC3A group was 0.83 ± 0.11 cm^3^ and 1.49 ± 0.18 cm^3^ in the SiHa-Vector group (Figure 7C). The average tumor weight in the SiHa-APOBEC3A group was 0.80 ± 0.35 g, and 1.57 ± 0.59 g in the SiHa-Vector group (Figure 7D). The representative hematoxylin-eosin staining pattern is shown in Figure 7B.

### APOBEC3A overexpression downregulates gene expression levels and inhibits multiple cancer-associated signaling pathways

**Figure 8:**
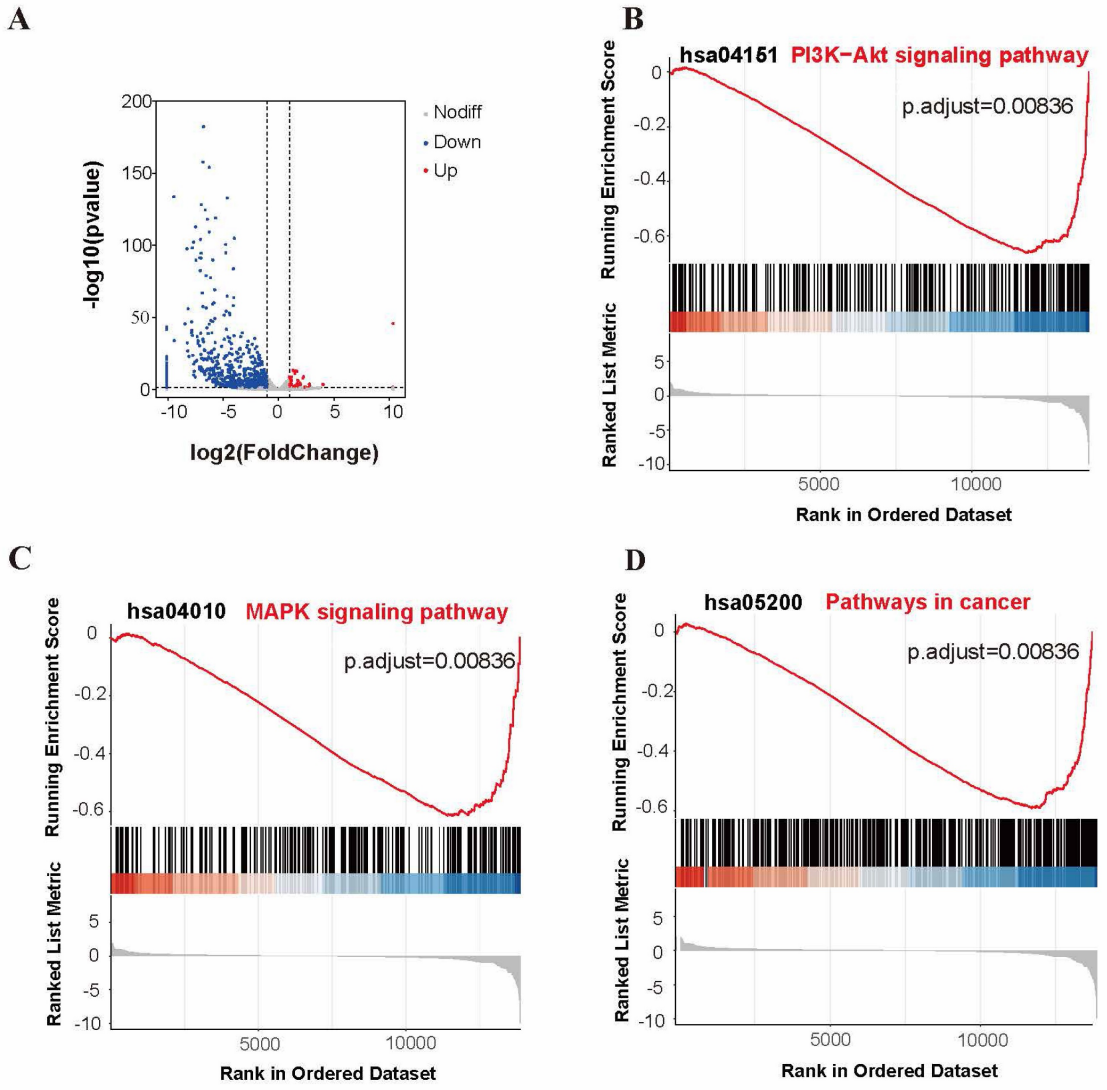
** APOBEC3A overexpression inhibits multiple cancer-associated signaling pathways.** (A) A volcano plot for differential gene expression between SiHa-APOBEC3A and SiHa-Vector. (B) Gene sets representing the signature of the PI3K-Akt signaling pathway. (C) Gene sets representing the signature of pathways in cancer. (D) Gene sets representing the signature of the MAPK signaling pathway.

**Figure 9:**
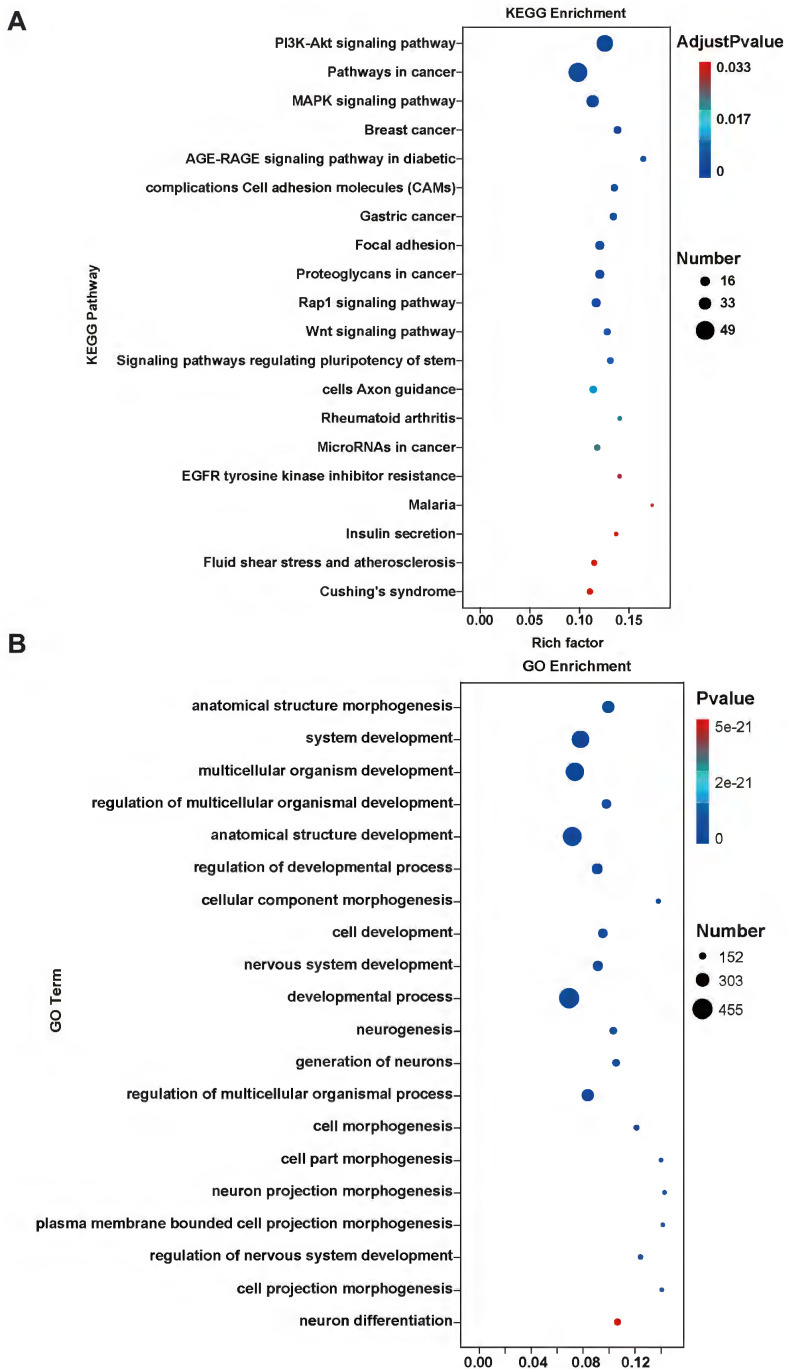
** KEGG and GO analysis of differentially expressed genes.** (A) KEGG pathway analysis of differentially expressed genes. (B) The GO analysis of differentially expressed genes.

We used RNA-Seq analysis to explore the pathways for APOBEC3A action in cervical cancer cells. There were 910 differentially expressed genes (fold change >2, *P*<0.05) (Figure 8A). Of these, 93.52% (851) were significantly downregulated in the SiHa-APOBEC3A cells compared with the SiHa-Vector cells. According to the Kyoto Encyclopedia of Genes and Genomes (KEGG) pathway analysis, differentially expressed genes were involved in “Pathways in cancer” (KEGG-hsa05200) and were enriched in the phosphatidylinositide 3-kinases-protein kinase B (PI3K-Akt) signaling pathway (KEGG-hsa04151) (Figure 9A). The significantly enriched gene ontology (GO) terms were related to biological processes, including anatomical structure morphogenesis (GO:0009653), system development (GO:0048731), and multicellular organism development (GO:0007275) (Figure 9B). The PI3K-Akt signaling pathway, the mitogen-activated protein kinase (MAPK) signaling pathway, and pathways in cancer were significantly downregulated by APOBEC3A (Figure 8B-8D). The top ten GSEA pathways are shown in Supplementary Figure S2.

## Discussion

APOBEC3A and pro-inflammatory factors IL-6 and NF-κB were more highly expressed in cervical cancer tissues than in adjacent normal tissues (Figure 1). APOBEC3A is a single-domain enzyme (i.e., only one zinc-dependent cytidine deaminase domain) and an influential restriction factor for HPV; APOBEC3 upregulation occurs throughout disease progression to suppress the infection 25, 26.

NF-κB activation upregulates the expression of APOBEC proteins in cervical cancer 27. Under the persistent stimulation of inflammatory factors, APOBEC3 is significantly upregulated in hepatocellular carcinoma 28. Our data support that HPV infection and higher inflammation levels in cervical cancer promote APOBEC3A expression.

We also observed an elevated level of UNG in cervical cancer tissues (Figure 1B). APOBEC cytosine deamidation is managed by the base excision repair (BER) system. UNG-initiated BER is the primary mechanism to counteract ingAPOBEC3-induced genomic mutations 29. Hence, the high level of APOBEC3A activates the BER system and upregulates UNG expression. However, the ratio of APOBEC3A to UNG is also increased in tumor tissues (Figure 1F), suggesting that the DNA damage-repair balance is disturbed in cervical cancer.

Somatic mutations are the basis of carcinogenesis in various cancers, and APOBEC3 is a significant source of somatic mutations. APOBEC3A-signature mutations are enriched in cervical cancer genome 16. However, our results indicated that although APOBEC3A is significantly upregulated in cancer tissues, it exerts an anti-cancer effect and correlates with better outcomes in cervical cancer. These findings are in consistent with previous studies 30, 31. But these previous related studies didn't explain this contradiction. Here, we presented and validated a hypothesis that may clarify this contradiction: Factors including HPV infection and high inflammation levels promote the expression of APOBEC3A in cervical cancer. However, the upregulation of APOBEC3A disturbs the DNA damage-repair balance because of its powerful mutagenic efficiency.

The APOBEC3A-mediated DNA damage exceeds the cellular tolerance, which promoting cancer cell apoptosis. Therefore, APOBEC3A conversely exerts an anti-cancer effect in cervical cancer. This hypothesis is also reported in renal cell carcinoma and biliary tract cancer 32, 33. The cancer evolution-development hypothesis holds that in virus-associated cancers, the virus and host cells undergo a mutation-selection-adaptation evolutionary process; the balance between the strength of mutagenesis and the tolerance of cells to genotoxicity is critical for cancer evolution and development 15. Steady-state APOBEC3A levels are very low in most cells because of tight regulation 34. Ectopic APOBEC3A expression may hinder cancer evolution at the mutagenesis stage because of its powerful mutagenic role.

APOBEC3A interacts with genomic DNA, resulting in genomic instability, DNA mutations, breaks, and damage 35. APOBEC3A functional polymorphisms increase APOBEC3A expression levels and significantly decrease the risk of renal cell carcinoma and biliary tract cancers. APOBEC3A overexpression exerts an anti-cancer effect by causing apoptosis in these cells 32, 33. Our study also showed that ectopically expressed APOBEC3A inhibits migration and invasion, induces DNA double strand break, and promotes apoptosis. RNA-seq indicated that most genes were downregulated, and several cancer-related signaling pathways were inhibited when APOBEC3A was upregulated. These findings suggest that APOBEC3A inhibits majority of genes transcription by promoting DNA/RNA damage. APOBEC3A exerts anti-tumor effects in cervical cancer is partly because the APOBEC3A-mediated DNA damage exceeds the cellular tolerance, thus promoting cancer cell apoptosis.

APOBEC3A overexpression causes cell-cycle arrest in a deaminase-dependent manner 36. The G2-phase checkpoint can prevent the initiation of mitosis and repair G2-phase and previously unrepaired DNA damage, primarily mediated by the ataxia-telangiectasia-mutated-and-Rad3-related kinase-checkpoint kinase 1 (ATR-Chk1) pathway. High APOBEC3A-expressing leukemia cells require the ATR-Chk1 checkpoint for survival, while blocking this pathway causes cell death 37. Our study also showed that ectopically expressed APOBEC3A caused cell-cycle arrest at the S-phase. Our study demonstrated that ectopically expressed APOBEC3A caused cell-cycle arrest in the S-phase and downregulated the G2 stage, suggesting that APOBEC3A inhibits the ATR-Chk1 pathway in cervical cancer cells, leading to cell death.

Our study has limitations. We focused on the effects of APOBEC3A on the biological behavior of cervical cancer cells and the relationship between APOBEC3A and outcomes. However, the somatic mutagenicity and antiviral effects of APOBEC3A are also essential for the occurrence and development of cervical cancer. First, HPV infection is a significant risk factor for cervical cancer. The SiHa cell line integrates a segment of the HPV16 genome (one to two copies per cell), while the HeLa cell line contains the HPV18 sequence (10 to 50 copies/cell) 38. APOBEC3A plays an important role in the innate immune response to viral infection by editing the viral genome. The relationship between APOBEC3A and HPV in cervical cancer development remains to be further investigated. Second, APOBEC3A has potent deamination activity in cervical cancer cell lines and is strongly associated with mutational burden in cervical cancers. The complex roles of APOBEC3A-induced somatic mutations in tumorigenesis and development should be investigated. Third, genetic polymorphisms may also influence individual susceptibility to cervical cancer and its outcome. However, several studies reported that genetic polymorphisms of APOBEC3A is not significantly associated with cervical cancer risk, cervical carcinogenesis and prognosis 31, 39-40. The association between the APOBEC3A polymorphism and cervical cancer needs to be further investigated.

## Conclusions

APOBEC3A is significantly overexpressed in cervical cancer, and higher expression of APOBEC3A is associated with better outcomes. APOBEC3A inhibits cell migration and invasion, arrests cells in S-phase, promotes cervical cancer cell apoptosis by inducing DNA damage that exceeds cellular tolerance, and inhibits several cancer-associated signaling pathways.

## Supplementary Material

Supplementary figures and tables.

## Abbreviations

APOBEC3A: Apolipoprotein B mRNA-editing enzyme catalytic 3A
ATR-Chk1: ataxia-telangiectasia-mutated-and-Rad3-related kinase-checkpoint kinase 1
CESC: cervical squamous cell carcinoma and endocervical adenocarcinoma
CIN: cervical intraepithelial neoplasia
GO: gene ontology
GSEA: Gene set enrichment analysis
HPV: human papillomavirus
HSILs: High-grade squamous intraepithelial lesions
IL-6: interleukin-6
KEGG: Kyoto Encyclopedia of Genes and Genomes
LSILs: low-grade squamous intraepithelial lesions
MAPK: mitogen-activated protein kinase
NF-κB: nuclear factor kappa-B
PI3K-Akt: phosphatidylinositide 3-kinases-protein kinase B
UNG: Uracil-DNA glycosylase

## References

[1] Singh D, Vignat J, Lorenzoni V (2023). Global estimates of incidence and mortality of cervical cancer in 2020: a baseline analysis of the WHO Global Cervical Cancer Elimination Initiative. Lancet Glob Health.

[2] Siegel RL, Miller KD, Wagle NS (2023). Cancer statistics, 2023. CA Cancer J Clin.

[3] Jiang D, Niu Z, Tan X (2023). The mortalities of female-specific cancers in China and other countries with distinct socioeconomic statuses: A longitudinal study. J Adv Res.

[4] Rahangdale L, Mungo C, O'Connor S (2022). Human papillomavirus vaccination and cervical cancer risk. Bmj.

[5] Chen L, Qiu X, Zhang N (2017). APOBEC-mediated genomic alterations link immunity and viral infection during human papillomavirus-driven cervical carcinogenesis. Biosci Trends.

[6] Farmanbar A, Firouzi S, Kneller R (2022). Mutational signatures reveal ternary relationships between homologous recombination repair, APOBEC, and mismatch repair in gynecological cancers. J Transl Med.

[7] Jarvis MC, Ebrahimi D, Temiz NA (2018). Mutation Signatures Including APOBEC in Cancer Cell Lines. JNCI Cancer Spectr.

[8] Zammataro L, Lopez S, Bellone S (2019). Whole-exome sequencing of cervical carcinomas identifies activating ERBB2 and PIK3CA mutations as targets for combination therapy. Proc Natl Acad Sci U S A.

[9] Henderson S, Chakravarthy A, Su X (2014). APOBEC-mediated cytosine deamination links PIK3CA helical domain mutations to human papillomavirus-driven tumor development. Cell Rep.

[10] de Oliveira GR, Carvalho PS, Vieira VC (2022). High APOBEC3B mRNA Expression Is Associated with Human Papillomavirus Type 18 Infection in Cervical Cancer. Viruses.

[11] Wei Z, Gan J, Feng X (2022). APOBEC3B is overexpressed in cervical cancer and promotes the proliferation of cervical cancer cells through apoptosis, cell cycle, and p53 pathway. Front Oncol.

[12] Fan Q, Huang T, Sun X (2021). HPV-16/18 E6-induced APOBEC3B expression associates with proliferation of cervical cancer cells and hypomethylation of Cyclin D1. Mol Carcinog.

[13] Liao X, Xia X, Su W (2022). Association of recurrent APOBEC3B alterations with the prognosis of gastric-type cervical adenocarcinoma. Gynecol Oncol.

[14] Zhu B, Xiao Y, Yeager M (2020). Mutations in the HPV16 genome induced by APOBEC3 are associated with viral clearance. Nat Commun.

[15] Liu W, Deng Y, Li Z (2021). Cancer Evo-Dev: A Theory of Inflammation-Induced Oncogenesis. Front Immunol.

[16] Ojesina AI, Lichtenstein L, Freeman SS (2014). Landscape of genomic alterations in cervical carcinomas. Nature.

[17] Cao GW (2017). Cancer Evo-Dev, a novel hypothesis derived from studies on hepatitis B virus-induced carcinogenesis. Hepatoma Res.

[18] Wakae K, Nishiyama T, Kondo S (2018). Keratinocyte differentiation induces APOBEC3A, 3B, and mitochondrial DNA hypermutation. Sci Rep.

[19] Liu W, Wu J, Yang F (2019). Genetic Polymorphisms Predisposing the Interleukin 6-Induced APOBEC3B-UNG Imbalance Increase HCC Risk via Promoting the Generation of APOBEC-Signature HBV Mutations. Clin Cancer Res.

[20] Deng Y, Du Y, Zhang Q (2014). Human cytidine deaminases facilitate hepatitis B virus evolution and link inflammation and hepatocellular carcinoma. Cancer Lett.

[21] Petljak M, Dananberg A, Chu K (2022). Mechanisms of APOBEC3 mutagenesis in human cancer cells. Nature.

[22] Xu F, Liu T, Zhou Z (2021). Comprehensive Analyses Identify APOBEC3A as a Genomic Instability-Associated Immune Prognostic Biomarker in Ovarian Cancer. Front Immunol.

[23] Argyris PP, Wilkinson PE, Jarvis MC (2021). Endogenous APOBEC3B overexpression characterizes HPV-positive and HPV-negative oral epithelial dysplasias and head and neck cancers. Mod Pathol.

[24] Tan X, He S, Han Y (2013). Establishment and characterization of clear cell renal cell carcinoma cell lines with different metastatic potential from Chinese patients. Cancer Cell Int.

[25] Vartanian JP, Guétard D, Henry M (2008). Evidence for editing of human papillomavirus DNA by APOBEC3 in benign and precancerous lesions. Science.

[26] Warren CJ, Xu T, Guo K (2015). APOBEC3A functions as a restriction factor of human papillomavirus. J Virol.

[27] Tilborghs S, Corthouts J, Verhoeven Y (2017). The role of Nuclear Factor-kappa B signaling in human cervical cancer. Crit Rev Oncol Hematol.

[28] Wang D, Li X, Li J (2019). APOBEC3B interaction with PRC2 modulates microenvironment to promote HCC progression. Gut.

[29] Serebrenik AA, Starrett GJ, Leenen S (2019). The deaminase APOBEC3B triggers the death of cells lacking uracil DNA glycosylase. Proc Natl Acad Sci U S A.

[30] Chen S, Li X, Qin J (2015). APOBEC3A possesses anticancer and antiviral effects by differential inhibition of HPV E6 and E7 expression on cervical cancer. Int J Clin Exp Med.

[31] Zhang M, Wei Z, Zhao H (2023). The role of APOBEC3A in cervical cancer development and progression: A retrospective study. Drug Discov Ther.

[32] Liu W, Ji H, Zhao J (2022). Transcriptional repression and apoptosis influence the effect of APOBEC3A/3B functional polymorphisms on biliary tract cancer risk. Int J Cancer.

[33] Tan X, Zheng S, Liu W (2021). Effect of APOBEC3A functional polymorphism on renal cell carcinoma is influenced by tumor necrosis factor-α and transcriptional repressor ETS1. Am J Cancer Res.

[34] Caval V, Bouzidi MS, Suspène R (2015). Molecular basis of the attenuated phenotype of human APOBEC3B DNA mutator enzyme. Nucleic Acids Res.

[35] DeWeerd RA, Németh E, Póti Á (2022). Prospectively defined patterns of APOBEC3A mutagenesis are prevalent in human cancers. Cell Rep.

[36] Landry S, Narvaiza I, Linfesty DC (2011). APOBEC3A can activate the DNA damage response and cause cell-cycle arrest. EMBO Rep.

[37] Green AM, Budagyan K, Hayer KE (2017). Cytosine Deaminase APOBEC3A Sensitizes Leukemia Cells to Inhibition of the DNA Replication Checkpoint. Cancer Res.

[38] Xu J, Liu H, Yang Y (2019). Genome-Wide Profiling of Cervical RNA-Binding Proteins Identifies Human Papillomavirus Regulation of RNASEH2A Expression by Viral E7 and E2F1. mBio.

[39] Revathidevi S, Manikandan M, Rao AK (2016). Analysis of APOBEC3A/3B germline deletion polymorphism in breast, cervical and oral cancers from South India and its impact on miRNA regulation. Tumour Biol.

[40] Castilha EP, Curti RRJ, de Oliveira JN (2023). APOBEC3A/B Polymorphism Is Not Associated with Human Papillomavirus Infection and Cervical Carcinogenesis. Pathogens.

