# Supplementary material for: APOBEC3A suppresses cervical cancer via apoptosis
Source: J Cancer. 2023 Oct 16;14(18):3429–43. doi: 10.7150/jca.89044 (PMC10647198; doi:10.7150/jca.89044)
Supplement: Supplementary file 1 — Supplementary figures and tables. [file jcav14p3429s1.zip › Supplemetary material/Supplemetary material.docx]

**Supplementary figure legend**

**Figure S1. Structure diagram of empty control plasmid and A3A overexpression plasmid.** (A) H8815-empty body; (B) H10695-A3A overexpression vector (Source: provided by OBIO TECHNOLOGY (SHANGHAI) CORP., LTD.)

**Figure S2. The top ten GSEA pathways.** AGE-RAGE: Advanced glycation end product- receptor for advanced glycation end product; MAPK: Mitogen-activated protein kinases; PI3K-Akt: Phosphatidylinositide 3-kinases-protein kinase B; Rap1: RAS-associated protein 1

**Supplementary Table S1.** **Baseline data of patients with cervical cancer.** FIGO: International Federation of Gynecology and Obstetrics; H-APOBEC3A: high expression level of APOBEC3A; L-APOBEC3A: low expression level of APOBEC3A.

**Supplementary Table S1.** Baseline data of patients with cervical cancer

| **Characteristic** | **L-APOBEC3A**  **（n = 14）** | **H-APOBEC3A**  **（n = 24）** | | **P Value** |
| --- | --- | --- | --- | --- |
| **Age (year)** | 49.43±7.968 | 46.58±7.667 | 0.284 | |
| **Histological grade — no. (%)** |  |  | 0.015 | |
| Poorly differentiated | 0 (0%) | 8 (33.3%) |  | |
| Moderately-Well differentiated | 14 (100%) | 16 (66.7%) |  | |
| **Tumor size（cm）** | 2.17±0.83 | 2.76±1.21 | 0.116 | |
| **FIGO stage — no. (%)** |  |  | 0.002 | |
| I | 14 (100%) | 10 (41.7%) |  | |
| II | 0 (0%) | 7 (29.2%) |  | |
| III | 0 (0%) | 7 (29.2%) |  | |
| IV | 0 (0%) | 0 (0%) |  | |
| **Lymph node metastasis — no. (%)** |  |  |  | |
| 0 | 14 (100%) | 18 (75.0%) | 0.041 | |
| 1 | 0 (0%) | 6 (25.0%) |  | |

**Supplementary Table S2.** **Baseline data of downloaded patients with cervical cancer.** FIGO: International Federation of Gynecology and Obstetrics; H-APOBEC3A: high expression level of APOBEC3A; L-APOBEC3A: low expression level of APOBEC3A.

**Supplementary Table S2.** Baseline data of downloaded patients with cervical cancer

| **Characteristic** | **L-APOBEC3A** | **H-APOBEC3A** | **P Value** |
| --- | --- | --- | --- |
|  | **（n = 37）** | **（n = 74）** |  |
| **Age (year)** | 46.86±11.078 | 47.14±12.822 | 0.91386 |
| **FIGO stage — no. (%)** |  |  | 0.08599 |
| I | 21 (56.76%) | 36 (48.65%) |  |
| II | 10 (27.03%) | 10 (13.51%) |  |
| III | 5 (13.51%) | 24 (32.43%) |  |
| Ⅳ | 1 (2.70%) | 4 (5.41%) |  |
| **Lymph node metastasis — no. (%)** |  |  | 0.05618 |
| 0 | 32 (86.49%) | 50 (67.57%) |  |
| 1 | 5 (13.51%) | 24 (32.43%) |  |

**Supplementary Table S3. Common clinical data of downloaded and hospital patients.** FIGO: International Federation of Gynecology and Obstetrics; H-APOBEC3A: high expression level of APOBEC3A; L-APOBEC3A: low expression level of APOBEC3A.

**Supplementary Table S3.** Common clinical data of downloaded and hospital patients

| **Characteristic** | **TCGA data**  **（n = 111）** | **Hospital data**  **（n = 38）** | **P Value** | |
| --- | --- | --- | --- | --- |
| **Age (year)** | 47.05±12.269 | 47.63±7.693 | 0.783854 | |
| **FIGO stage — no. (%)** |  |  | 0.3712（χ² =3.1357） | |
| I | 57（51.35%） | 24（63.16%） | |  |
| II | 20（18.01%） | 7（18.42%） | |  |
| III | 29（26.13%） | 7（18.42%） | |  |
| Ⅳ | 5（4.50%） | 0（0%） |  | |
| **Lymph node metastasis — no. (%)** |  |  | 0.2821（χ² =1.157） | |
| 0 | 82（73.87%） | 32（84.21%） | |  |
| 1 | 29（26.13%） | 6（15.79%） | |  |
