# Supplementary figures and images for: APOBEC3A suppresses cervical cancer via apoptosis
Source: J Cancer. 2023 Oct 16;14(18):3429–43. doi: 10.7150/jca.89044 (PMC10647198; doi:10.7150/jca.89044)

**A**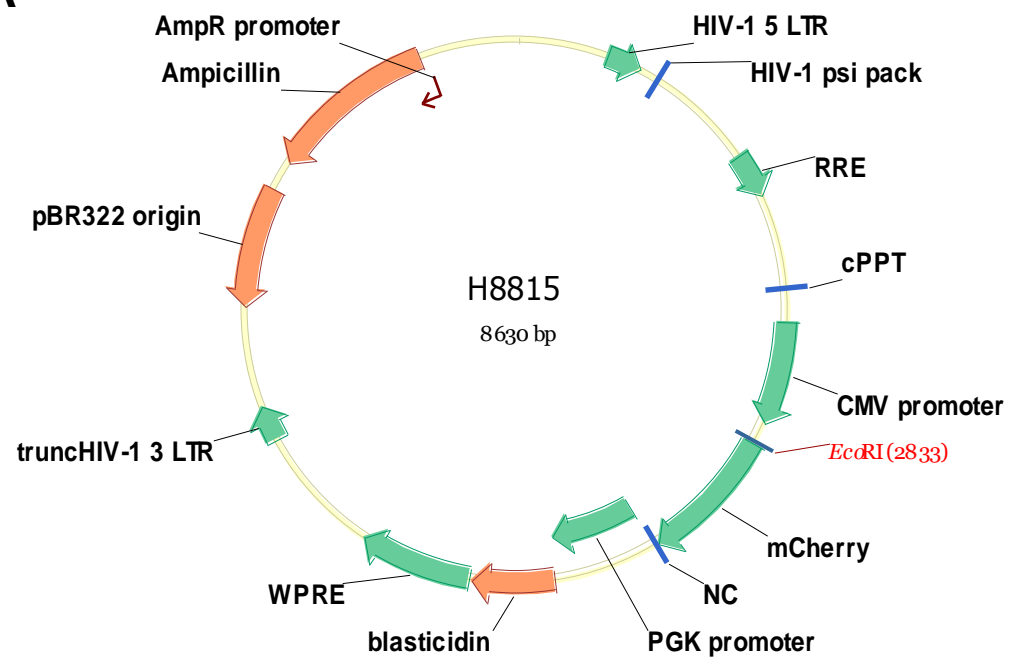**B**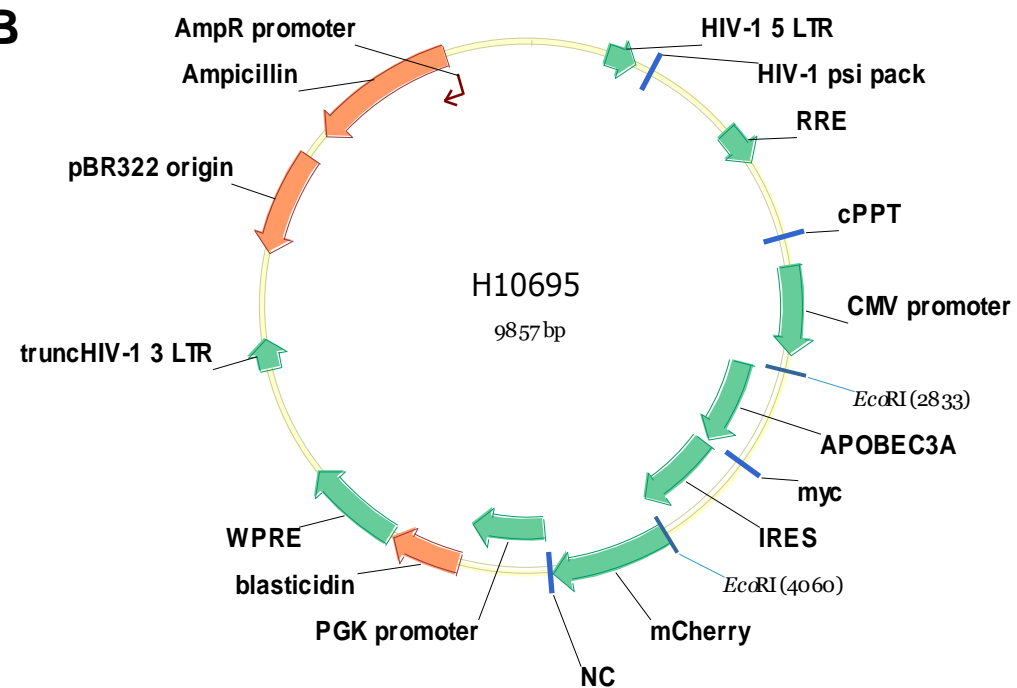

Supplement: Supplementary file 1 — Supplementary figures and tables. [file jcav14p3429s1.zip › Supplemetary material/Supplementary Figure S1.pdf]

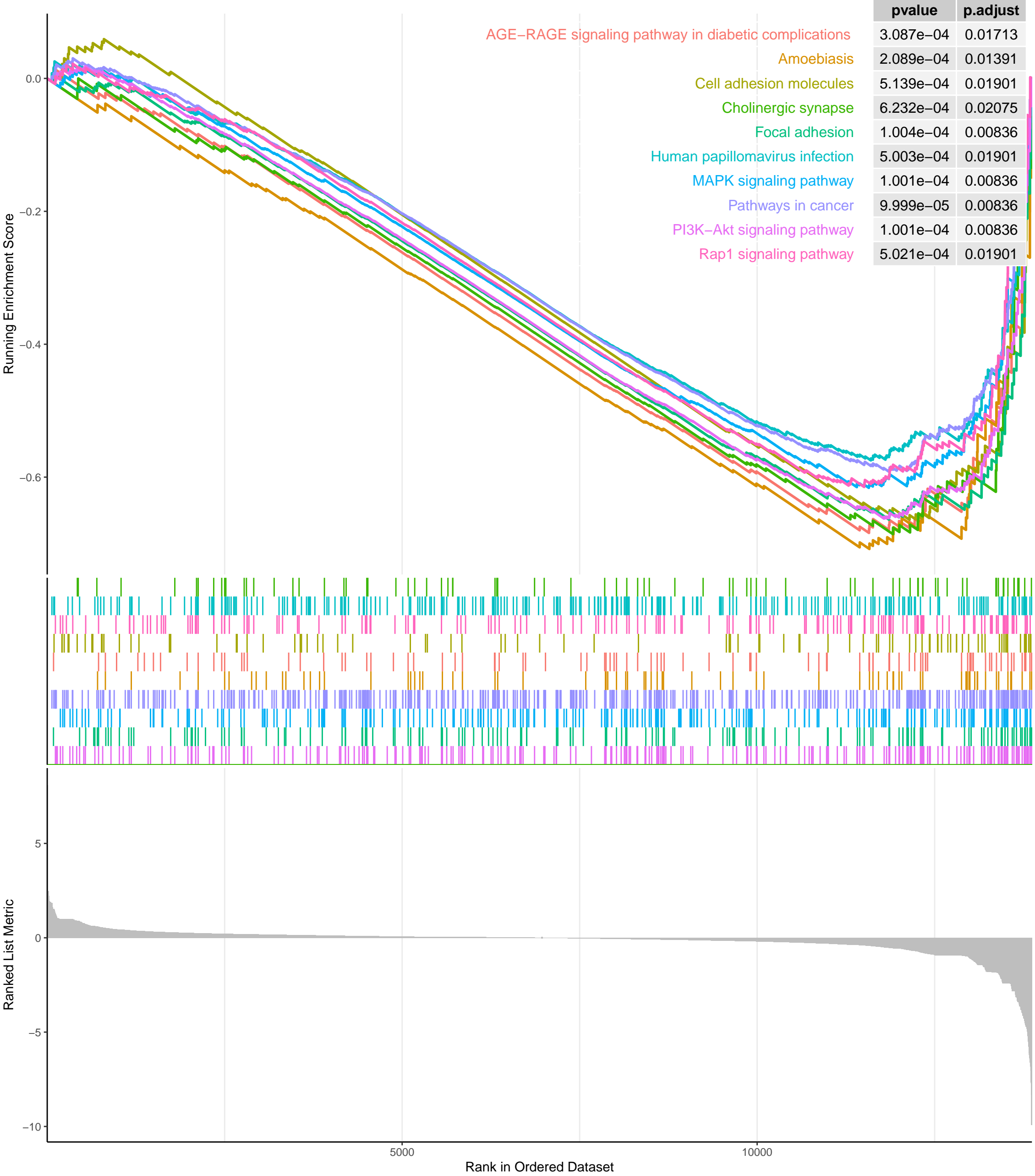

Supplement: Supplementary file 1 — Supplementary figures and tables. [file jcav14p3429s1.zip › Supplemetary material/Supplementary Figure S2.pdf]
